# Pyelonephritis and Bacteremia from *Lactobacillus delbrueckii*

**DOI:** 10.1155/2012/745743

**Published:** 2012-09-29

**Authors:** Kevin M. DuPrey, Leon McCrea, Bonnie L. Rabinowitch, Kamran N. Azad

**Affiliations:** ^1^Department of Family Medicine, Crozer-Keystone Health System, Springfield, PA 19064, USA; ^2^Department of Infectious Disease, Crozer-Keystone Health System, Springfield, PA 19064, USA; ^3^Quest Diagnostics Nichols Institute, San Juan Capistrano, CA 92675, USA

## Abstract

Lactobacilli are normal colonizers of the oropharynx, gastrointestinal tract, and vagina. Infection is rare, but has been reported in individuals with predisposing conditions. Here we describe the case of a woman with pyelonephritis and bacteremia in which *Lactobacillus delbrueckii* was determined to be the causative agent.

## 1. Introduction

Lactobacilli are ubiquitous commensal gram positive rods that colonize the mucosal surfaces of the oropharynx, gastrointestinal tract, and vagina. Although rare, reports have shown lactobacilli to cause bacteremia [1], subacute endocarditis [1, 2], urinary tract infections [3, 4], meningitis [5], chorioamnionitis [2], endometritis, abscesses, and dental caries [1]. The true prevalence of lactobacilli infection is likely underreported in the medical literature as the bacterium is typically regarded as commensal or contamination when identified. To our knowledge, this is the first case of pyelonephritis and bacteremia caused by *L. delbrueckii*. We review the literature to identify key risk factors for *Lactobacillus* bacteremia from a renal source including: urolithiasis, diabetes, cancer, and recent use of certain antibiotics. Here we describe our case to demonstrate significant illness in an individual with multiple predisposing conditions.

## 2. Case Report

A 68-year-old woman presented to the emergency department with fever, chills, nausea, and vomiting. She was diagnosed with a urinary tract infection by urinalysis and discharged home on ciprofloxacin 500 mg twice daily. She returned the following day with persistent fevers, chills, nausea, vomiting, and new onset confusion, diaphoresis, abdominal and left-sided flank pain. Her significant past medical history included uncontrolled type 2 diabetes mellitus, hypothyroidism, chronic obstructive pulmonary disease (COPD), and tobacco use.

On examination, the patient was febrile (39.4°C), hypotensive (75/20 mmHg), tachycardic (pulse 104 beats/minute), tachypneic (respiratory rate 24/minute), and hypoxic (oxygen saturation of 78% on room air). She was confused and appeared fatigued. Left-sided costovertebral angle tenderness and suprapubic tenderness were noted. Laboratory data showed a normal white cell count (of 5.0 × 10^9^/L). Blood glucose was elevated at 362 mg/dL (normal 70–110 mg/dL). Blood urea nitrogen and creatinine were elevated at 28 mg/dL (normal 10–20 mg/dL) and 1.7 mg/dL (normal 0.6–1.1 mg/dL), respectively. Urinalysis showed a pH of 5 (normal 5–8), negative nitrites, 3 plus leukocyte esterase/high powered field (hpf), 20–30 white blood cell (WBC)/hpf and glucose of 1000 mg/dL.

The patient was admitted to the intensive care unit with suspected sepsis from a renal source. Intravenous antibiotics were initiated including vancomycin 1 gram every 24 hours and cefepime 2 grams every 24 hours. Imaging with retroperitoneal ultrasound and abdominopelvic computerized tomography showed a partially obstructing 6 mm left ureteral calculus with mild left hydronephrosis. Cystoscopy was performed with placement of a double J stent with stone manipulation into the kidney. Urine cultures from her original emergency department visit and subsequent hospitalization of both clean catch and straight catheterized specimens demonstrated *Lactobacillus* species of >100,000 colony forming units (CFU)/mL.

Blood cultures grew gram positive rods in both aerobic and anaerobic bottles in four out of four sets. Recovered blood-culture isolate revealed *Lactobacillus delbrueckii*. Susceptibility testing was performed and minimum inhibitory concentration (MIC) in mcg/mL was interpreted and reported for ampicillin (≤0.120, susceptible), clindamycin (≤0.500, susceptible), erythromycin (≤0.250, susceptible), gentamicin (≤2, susceptible), penicillin (≤0.060, susceptible), and vancomycin (≤0.250, susceptible) according to Clinical and Laboratory Standards Institute (CLSI) guidelines.

Following identification of *Lactobacillus* species, vancomycin and cefepime were discontinued and the patient was started on ampicillin 2 grams IV every 6 hours. All subsequent blood and urine cultures were negative. The patient continued to improve throughout hospitalization and was discharged to a skilled nursing facility one week following admission with close out-patient follow up. She completed a 2-week course of ampicillin then underwent ureteroscopy and holmium laser lithotripsy of the caculi. She has been stable to date, 9 months following presentation.

## 3. Discussion

Lactobacilli are ubiquitous commensal gram positive rods that colonize the mucosal surfaces of the oropharynx, gastrointestinal tract, and vagina. They are anaerobic or facultatively anaerobic and ferment glucose to produce lactic acid. Although rare, lactobacilli have been reported to cause infection in susceptible individuals. Reports have shown lactobacilli to cause bacteremia [1], subacute endocarditis [1, 2], urinary tract infections [3, 4], meningitis [5], chorioamnionitis [2], endometritis, abscesses, and dental caries [1].

Debate exists regarding the significance of lactobacilli identified in clinical specimens. The true prevalence of *Lactobacilli* infection is likely underreported in the medical literature as the bacterium is typically regarded as commensal or contamination when identified. The mortality rate associated with *Lactobacillus* bacteremia alone is thought to be low [6]. Husni et al. showed that *Lactobacillus* bacteremia cleared in 98% (*n* = 45) of patients after appropriate treatment. However, literature has shown 1 year mortality following *Lactobacillus* bacteremia ranging from 48% to 69% [7, 8]. The cause of death was attributed to underlying disease rather than *Lactobacillus* bacteremia in most cases. Thus, lactobacilli are uncommon pathogens but can serve as a marker for severe and rapidly fatal conditions.

Bacteremia from a renal source due to *Lactobacillus* species is rare. To our knowledge, this is the first case of sepsis from a renal source caused by *L. delbrueckii*. Our literature review revealed one case of a urinary tract infection caused by *L. delbrueckii* [3]. Five other cases of *Lactobacillus* bacteremia from a renal source were found in addition to our case as shown in Table 1. Cases involved mostly women in the 6th and 7th decades of life. Risk factors for *Lactobacillus* bacteremia from a renal source include urolithiasis, use of certain antibiotics, and immunocompromising conditions such as diabetes and cancer [4, 9–12].

The pathogenesis of *Lactobacillus* bacteremia from a renal source is likely multifactoral. In immunocompetent patients, lactobacilli are thought to stimulate the local and systemic immune response and enhance mucosal function [13]. However, when mucosal function is compromised in certain clinical settings, at-risk individuals may be predisposed to *Lactobacillus* infection. Urolithiasis can cause urinary stasis which increases the risk of urinary tract infection [14]. Uncontrolled diabetes can lead to vascular compromise, renal papillary necrosis, and nephropathy which may contribute to increased risk of infection [15]. Use of certain antibiotics may select for *Lactobacillus* species and cause infection in susceptible individuals. Studies have shown almost uniform resistance to trimethoprim-sulfamethoxazole [16] and ciprofloxacin [17], with general resistance to vancomycin [7], metronidazole [1], and third generation cephalosporins [16]. Although exceedingly rare, use of probiotics has been associated with development of *Lactobacillus* bacteremia [8, 18]. While our patient denied taking probiotic supplements, she did note consuming 1 container of yogurt daily. By definition, the production of yogurt depends on the interaction between 2 specific bacterial subspecies: *Lactobacillus delbrueckii* ssp. *bulgaricus* and *Streptococcus salivarius* ssp. *thermophilus* [19]. Thus, it is possible that our patient developed vaginal colonization with *L. delbrueckii* from consuming yogurt and developed a urinary tract infection, pyelonephritis, and sepsis in the setting of urinary stasis with multiple predisposing factors. Although this theory is unable to be proven, we asked our patient to avoid yogurt and probiotics in the future as a precautionary measure.

In our case, laboratory analysis supports *Lactobacillus delbruekii* as the cause of infection. The acidic pH (5.0) on multiple urinalyses was consistent with infection from the bacterium since in *Lactobacillus* species, a large part of the carbon is transformed in organic acids [20]. All urinalyses showed negative nitrite testing consistent with the bacterium as lactobacilli do not reduce nitrate. Positive mid-stream voided, straight catheter and renal pelvis urine specimens demonstrated pure *Lactobacillus* species in significant quantities. Four out of four aerobic and anaerobic blood cultures grew *Lactobacillus* species in significant quantities. Analysis of the blood culture isolate was confirmed to be *L. delbrueckii* by four databases including Silva-Living Tree, BLAST NCBI (Basic Local Alignment Search Tool of the National Center for Biotechnology Information), RDP (Ribosomal Database Project), and SmartGene databases to confirm gene sequencing. All data were consistent with the identification and species differentiation (by guidelines of 0.8% separation to reach species differentiation). Despite numerous urine and blood specimens analyzed, no concomitant organisms were identified. Although further analysis of urine cultures to identify *Lactobacillus* species would have been helpful in confirming *L. delbrueckii* as the causative agent, they were not sent as the results would not have changed clinical management at the time.

## 4. Conclusion

In conclusion, we report a case of a patient with pyelonephritis and bacteremia in which *L. delbrueckii* was determined to be the causative agent. Although lactobacilli are uncommon pathogens, this case demonstrates significant illness in an individual with multiple predisposing conditions. Thus, we suggest prompt evaluation and appropriate treatment with identification of lactobacilli in individuals with risk factors including urolithiasis, recent antibiotic use and/or immunologically compromising conditions such as diabetes or cancer.

## Figures and Tables

**Table 1:** Cases of *Lactobacillus* bacteremia from a renal source.

Age/Sex^1^	Diabetes	Urolithiasis	Cancer	Abx^2^	Survival	Organism
52 M		X			X	Species unknown [10]
66 F	X	X		X	X	*Lactobacillus gasseri* [4]
51 F	X		X	X		Species unknown [11]
59 F	X	X	X		X	*Lactobacillus jensenii* [9]
63 F		X	X		X	*Lactobacillus acidophilus *[12]
68 F	X	X		X	X	*Lactobacillus delbrueckii* ^ 3^

^1^Age in years, M: male, F: female.

^2^Recent antibiotic use including: cefotaxime, vancomycin, or ciprofloxacin.

^3^Author's case report.
